# Longitudinal trends of peripheral blood counts in polycythaemia vera and essential thrombocythemia patients in the UK

**DOI:** 10.1002/jha2.519

**Published:** 2022-06-29

**Authors:** Lewis Carpenter, Patrick Rockenschaub, Grace B. Hatton, Sofia D'Abrantes, Edward Sims, Nicholas Scott‐Ram, Aurélie Ducès, Gabrielle Emanuel, Adam J. Mead, Mark W. Drummond, Nadezda Lipunova

**Affiliations:** ^1^ Sensyne Health Oxford Science Park Oxford UK; ^2^ Bristol Myers Squibb Ltd Uxbridge Business Park Uxbridge UK; ^3^ Medical Research Council Weatherall Institute of Molecular Medicine John Radcliffe Hospital, Headington Oxford UK; ^4^ Department of Haemato‐Oncology Beatson West of Scotland Cancer Centre Glasgow UK

**Keywords:** epidemiology, essential thrombocythaemia, polycythaemia vera

## Abstract

There is sparse evidence of how well haematological targets are met in practice for essential thrombocythemia (ET) and polycythaemia vera (PV) patients. Patient data was collected between 2008 and 2020 from two UK NHS Trusts for ET and PV patients. Longitudinal changes in peripheral blood counts, including the proportion of patients meeting peripheral blood count remission, was modelled. Relative risk of cardiovascular‐related events for patients achieving remission within 3‐months was estimated. A total of 620 ET and 429 PV patients were analysed. For high‐risk patients, haematological parameters decreased in the first months of observation then stabilised within normal reference ranges until year 5. Total time spent in peripheral blood count remission was 39.2% for ET and 29.1% for PV. A lower proportion of ET patients reached target platelet counts (48.3%) compared to WBC (79.1%), whilst PV patients were less likely to reach target haematocrit levels (56.9%) compared to platelets (77.3%) or WBC (74.6%). There was no statistically significant association between reaching target blood counts within 3‐months and cardiovascular risk. Complete haematological remission remains a challenging target in managing PV and ET, however this study was unable to show statistically‐significant evidence that this was associated with increased risk of cardiovascular events.

## 1 INTRODUCTION

Essential thrombocythemia (ET) and polycythaemia vera (PV) are myeloproliferative neoplasms (MPNs) [1], characterised by abnormal proliferation of one or more cell lineages in the bone marrow [2]. Clinical manifestations of ET and PV include increased risk of thrombosis, as well as bleeding, splenomegaly, microcirculatory complications, and disease progression into secondary myelofibrosis and/or acute myeloid leukaemia (AML) [3].

Routinely measured haematological parameters are a crucial component of the World Health Organisation (WHO) diagnostic criteria for ET and PV [2, 4], and have a role in disease prognosis and monitoring. Clinically, ET is characterised by an excess platelet count, whereas PV is broadly defined by abnormally raised haemoglobin, haematocrit, and/or red cell mass [2, 4, 5].

Studies have highlighted the validity of using platelet count as a therapeutic target in ET and haematocrit in PV, primarily with the use of hydroxyurea treatment in lowering platelet counts, haematocrit and thrombotic risk [6, 7, 8, 9]. Treatment is aimed at lowering risk from vascular complications, whilst minimising the risk of disease progression to myelofibrosis or AML [10]. For high‐risk patients (age≥ 60 and/or with a history of thrombotic events), the use of cytoreductive therapeutics, anti‐platelet drugs, and venesection are standard of care for PV, whilst anti‐platelet drugs and cytoreductive treatments are used in patients with ET [3].

Whilst there is significant debate in the literature about what haematological values clinicians should aim for in routine care [10], it is broadly accepted that a target haematocrit < 45% significantly reduces the risk of thrombotic events in PV patients [11], and a target platelet count ≤400 × 10^9^/L as is associated with reduced risk of thrombotic and haemorrhagic events in ET [10]. Additionally, a leukocyte target of ≤10 × 10^9^/L is also recommended for both ET and PV. These thresholds are based on consensus expert opinion by the European LeukemiaNet (ELN) and International Working Group‐Myeloproliferative Neoplasms Research and Treatment (IWG‐MRT) [12].

Although studies have examined the significance of achieving haematological remission in both ET [13, 14, 15] and PV [16] on thrombotic risk outside the clinical trial setting, these studies have typically focused on sub‐populations exposed to specific medications or have been restricted by shorter lengths of follow‐up. This study therefore aims to examine the longitudinal progression of key haematological parameters in high‐risk ET and PV patients treated in a real‐world setting and examine the extent to which patients can achieve and sustain haematological target values over the course of their disease.

## 2 METHODS

### 2.1 Data source

The data were collected in Chelsea & Westminster Hospital NHS Foundation Trust (CW) and Oxford University Hospitals NHS Foundation Trust (OUH). We obtained anonymised patient‐level electronic health records (EHR) between years 2008–2020 for CW, and 2012–2020 for OUH. Both data sources were used to derive one population for the study.

### 2.2 Study population

Analyses were restricted to adult patients (aged 18 years and older) who were diagnosed with ET (ICD‐10 code: D47.3) or PV (ICD‐10 code: D45) during the study period. Patients entered the cohort on the date of their first ever recorded diagnosis with ET or PV. Patients were excluded if they had a diagnosis of MF or AML prior to their first observed diagnosis of ET or PV. Where JAK2 information for PV patients was recorded and negative, patients were excluded from the analysis. Patients were censored at the earliest of either: date of death; date of recorded transformation to MF or AML; two years after the last record of any clinical event; or the time of the data extraction (20^th^ December 2020). Given the high level of attrition beyond 5‐years, longitudinal analysis was restricted to 5‐years from cohort entry. Patients with less than 5‐years follow‐up were included in all analyses.

### 2.3 Variables

Age at first ET or PV diagnosis, sex, self‐reported ethnicity (categorised as White, Asian, Black, or Other), recorded diagnoses prior to or at cohort entry (in accordance with the International Classification of Diseases, tenth revision [ICD‐10]), and information on blood counts (red blood cells (RBC), platelets, haematocrit (HCT), haemoglobin (Hb), and white blood cells [WBC]) were included for each patient. In addition, the Charlson Comorbidity Index (CCI) was calculated for each patient [17]. Patients were defined as high risk if they were ≥ 60 years at cohort entry and/or had a history of haemorrhagic or thrombotic events (supplemental data in Table 1 for a list of detailed definitions using ICD10 codes). Information on genetic testing relevant to MPNs (*JAK2, MPL*, and *CALR*) was not well recorded in the routine patient records and excluded from the main analysis.

Peripheral blood count remission was defined based on the ELN revised response criteria: haematocrit < 45% (PV only), platelets ≤ 400 × 10^9^ / L, and WBC < 10 × 10^9^ / L. Absence of leukoerythroblastosis − which is an additional criterion for remission in ET − could not be ascertained from our data and was excluded from the analysis. The occurrence of the following clinical outcomes was ascertained over the course of the study: all‐cause mortality, thrombosis, haemorrhage, and cardiovascular disease (supplemental data in Table 1). Incidence of any of these conditions was defined as the earliest recording of a corresponding ICD‐10 code as the primary reason for hospital admission.

### 2.4 Statistical analysis

The patient characteristics at cohort entry were summarised for all patients using the median and interquartile range (IQR; continuous variables) and counts and percentages (categorical variables). To account for potential data entry delays, a window of 30‐days before or after their initial MPN diagnosis was chosen. If more than one value of a laboratory measurement were recorded within ±30 days, the average of those values was chosen.

For high‐risk ET and PV patients, longitudinal trends over the patients’ follow‐up period were analysed using linear mixed models with a Gaussian error distribution and a random intercept and time slope per patient, modelling all components of the FBC separately (RBC, platelets, HCT, Hb, and WBC) from 30 days before their initial MPN diagnosis (to include any values that led to the diagnosis of MPN) until the end of patients’ follow‐up. Before modelling, FBC results were transformed using a Box‐Cox power transformation. Non‐linear trends over time were accounted for using linear splines, with knots at 1‐month and 1‐year to account for systematic differences in blood test results around the initial MPN diagnosis. All models were adjusted for age, sex, CCI, and whether the index diagnosis was the main reason for hospital admission. Marginal estimates and corresponding 95% confidence intervals (CI) from linear models were used to represent time trends graphically and provide average values at cohort entry, after 1‐year, and after 5‐years. Marginal estimates represented the expected average values for a typical 60‐year‐old female (ET) or male (PV) patient. Additionally, 95% confidence intervals based on residual model variation estimated plausible ranges within which measurements of a single patient could be expected to fluctuate over the course of follow‐up.

The proportion of high‐risk patients that achieved peripheral blood count remission at least once during follow‐up was calculated. Full peripheral blood count remission was defined as meeting all ELN thresholds defined above. Each haematological marker was then examined individually (HCT, platelets and WBC for PV and platelets and WBC for ET). The time spent in and out of remission for each patient overall and for haematocrit, platelets, and WBC individually was plotted. Finally, we estimated the association between achieving full or partial peripheral blood count remission within 3 months of cohort entry and the risk of developing included clinical outcomes using Cox regression models, adjusting for age, sex, and CCI.

## 3 RESULTS

A total of 620 ET and 429 PV patients were included across both data sources (Table 1).

**TABLE 1:** Baseline characteristics of ET and PV patients at time of index diagnosis

	ET (*N* = 620)	Missing (%)	PV (*N* = 429)	Missing (%)
**Demography**				
Age at cohort entry (median [IQR])	67.4 (51.2, 79.0)	0.0%	65.6 (54.2, 75.4)	0.0%
Age at cohort entry (%)		0.0%		0.0%
<60 years	227 (36.6%)		157 (36.6%)	
≥60 years	393 (63.4%)		272 (63.4%)	
Sex (%)		0.0%		0.0%
Male	251 (40.5%)		292 (68.1%)	
Female	369 (59.5%)		137 (31.9%)	
Ethnicity (%)		19.0%		21.0%
White	421 (83.9%)		294 (86.7%)	
Asian	37 (7.4%)		21 (6.2%)	
Black	11 (2.2%)		9 (2.7%)	
Other	33 (6.6%)		15 (4.4%)	
**Comorbidities**				
H/O haemorrhage or thrombosis (%)	109 (17.6%)	5.5%	53 (12.4%)	26.6%
CCI (%)		5.5%		26.6%
0	224 (38.2%)		153 (48.6%)	
1—2	264 (45.1%)		132 (41.9%)	
≥3	98 (16.7%)		30 (9.5%)	
**Blood Counts**				
Haematocrit (%) (median [IQR])	35.1 (30.3, 39.7)	14.2%	47.5 (43.4, 51.6)	15.9%
Haemoglobin (g/dl) (median [IQR])	11.6 (9.8, 13.1)	14.2%	15.6 (13.7, 17.2)	15.9%
Platelets (x 10^9^/L) (median [IQR])	524.0 (382.3, 681.9)	14.4%	328.7 (222.1, 496.0)	15.9%
RBC count (x 10^12^/L) (median [IQR])	3.8 (3.3, 4.4)	14.4%	5.5 (4.8, 6.1)	15.9%
WBC count (x 10^9^/L) (median [IQR])	10.3 (7.8, 12.8)	14.4%	8.9 (7.0, 12.3)	15.9%
**Risk Stratification**				
Low risk	203 (32.7%)		141 (32.9%)	
*Of which had FBC results recorded*	*183 (90.1%)*		*129 (91.5%)*	
High risk	417 (67.3%)		288 (67.1%)	
*Of which had FBC results recorded*	*374 (89.7%)*		*262 (91.0%)*	

Abbreviations: CCI, Charlson Comorbidity Index; ET, essential thrombocythaemia; H/O, history of; IQR, interquartile range; PV, polycythaemia vera; RBC, red blood cells; WBC, white blood cells; FBC, full blood count.

Approximately two‐thirds of ET patients were female (59.5%) with a median age of 67.4 years, whilst 31.9% of PV patients were female with a median age of 66.7 years at cohort entry. Both cohorts were predominately white, and levels of comorbidity were increased in the ET cohort relative to PV. ET patients exhibited elevated median levels of platelets of 524.0 × 10^9^/L (IQR 382.3‐681.9) and WBCs at 10.3 × 10^9^/L (IQR 7.8‐12.8) at cohort entry. Patients with PV showed high median levels of haematocrit (47.5%, IQR 43.4‐51.6) and haemoglobin (15.6 g/dl, IQR 13.7‐17.2), and elevated levels of RBCs (5.5 × 10^12^/L (IQR 4.8‐6.1). Blood counts were generally well recorded around cohort entry, with approximately 85% of patients having at least one value within ±30 days of the estimated index date. A total of 109 (17.6%) ET patients and 53 (12.4%) PV patients had a recorded history of haemorrhage or thrombosis and 417 (67.3%) and 288 (67.1%) patients were considered as high‐risk patients and included in the further analysis.

### 3.1 Longitudinal trends of FBC results in high‐risk patients

**FIGURE 1:**
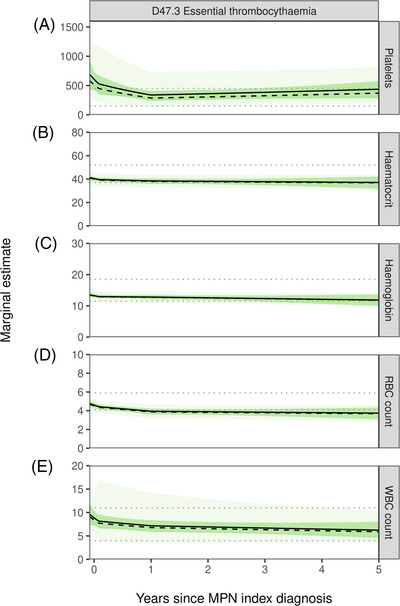
Marginal estimate plots for platelets, haematocrit, haemoglobin, RBC and WBC count in high‐risk ET patients. (A) Platelets(10^9^/L), (B) haematocrit (%), (C) haemoglobin (g/dl), (D) RBC count (10^12^/L) and e) WBC count (10^9^/L) for a 60‐year‐old, female (solid black line) and male (dashed black line) ET patient. The dark green shaded area represents the 95% confidence intervals around the marginal estimates, whilst the light green shaded area represents the 95% confidence intervals of the models’ residual variation. The grey dotted lines represent the upper and lower normal reference ranges for the respective blood marker (ET – essential thrombocythaemia, RBC – red blood cell count, WBC – white blood cell count)

**FIGURE 2:**
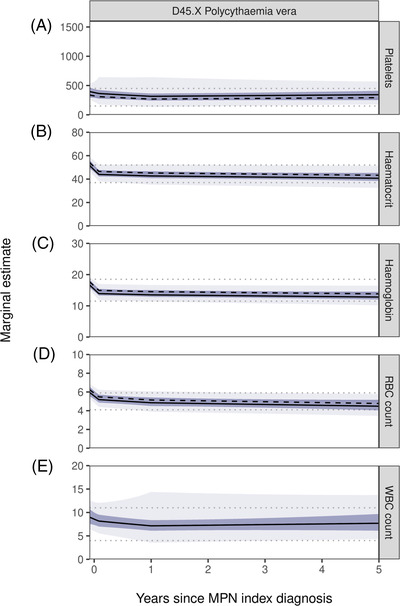
Marginal estimate plots for platelets, haematocrit, haemoglobin, RBC and WBC count in high‐risk PV patients. (A) platelets(10^9^/L), (B) haematocrit (%), (C) haemoglobin (g/dl), (D) RBC count (10^12^/L) and (E) WBC count (10^9^/L) for 60‐year‐old, female (solid black line) and male (dashed black line) PV patient. The dark green shaded area represents the 95% confidence intervals around the marginal estimates, whilst the light green shaded area represents the 95% confidence intervals of the models’ residual variation. The grey dotted lines represent the upper and lower normal reference ranges for the respective blood marker (PV – polycythaemia vera, RBC – red blood cell count, WBC – white blood cell count)

Of the 417 high‐risk ET patients and 288 high‐risk PV patients, 374 (90%) and 262 (91%) had at least one blood count recorded respectively over the 5‐years and were included in the longitudinal analysis (Table 1). Following patients over time, higher average blood count measurements were observed in both diseases close to cohort entry which tended to stabilise at a constant level after 3 to 12 months (Table 2 and Figures 1 and 2).

**TABLE 2:** Marginal estimates for each blood count per MPN for males and females

Laboratory marker	*N*	Sex	Index date Mean (95% CI)	Year 1 Mean (95% CI)	Year 5 Mean (95% CI)
**ET (*N* = 374)**					
HCT (%)	13,920	Male	40.1 (38.3–41.8)	38.1 (35.9–40.3)	36.8 (31.8–41.8)
		Female	40.4 (38.7–42.2)	38.5 (36.2–40.7)	37.1 (32.1–42.2)
Hb (g/dl)	14,488	Male	13.2 (12.6–13.8)	12.8 (12.0–13.5)	11.8 (10.0–13.7)
		Female	13.2 (12.6–13.9)	12.8 (12.0–13.6)	11.9 (10.0–13.7)
Platelets (x 10^9^/L)	11,170	Male	512.2 (422.8–620.6)	286.1 (237.1–345.2)	369.9 (281.0–486.9)
		Female	604.6 (497.0–735.5)	337.7 (278.0–410.1)	436.6 (330.6–576.7)
RBC (x 10^12^/L)	11,225	Male	4.5 (4.3–4.8)	3.9 (3.6–4.2)	3.7 (3.1–4.3)
		Female	4.6 (4.3–4.8)	3.9 (3.7–4.2)	3.7 (3.1–4.3)
WBC (x 10^9^/L)	11,231	Male	8.4 (7.2–9.7)	6.8 (5.8 –7.9)	5.9 (4.6–7.6)
		Female	8.9 (7.6–10.3)	7.2 (6.1–8.4)	6.2 (4.8–8.0)
**PV (*N* = 262)**					
HCT (%)	9677	Male	50.3 (48.9–51.8)	45.2 (43.7–46.7)	43.2 (41.2–45.2)
		Female	47.8 (46.0–49.7)	42.7 (40.8–44.5)	40.7 (38.4–43.0)
Hb (g/dl)	9788	Male	16.3 (15.8–16.8)	14.5 (14.0–15.0)	13.8 (13.1–14.5)
		Female	15.3 (14.7–16.0)	13.5 (12.9–14.2)	12.8 (12.0–13.6)
Platelets (x 10^9^/L)	8947	Male	323.8 (290.7–360.6)	268.8 (242.1–298.5)	292.4 (252.1–339.3)
		Female	379.9 (328.4–439.6)	315.4 (273.6–363.7)	343.2 (287.4–409.8)
RBC (x 10^12^/L)	8981	Male	5.9 (5.6–6.1)	5.1 (4.9–5.4)	4.8 (4.4–5.2)
		Female	5.6 (5.2–5.9)	4.9 (4.5–5.2)	4.5 (4.0–4.9)
WBC (x 10^9^/L)	8991	Male	8.6 (7.7–9.6)	7.2 (6.4–8.0)	7.7 (6.3–9.4)
		Female	8.6 (7.3–10.0)	7.1 (6.1–8.4)	7.7 (6.1–9.7)

Abbreviations: CCI, Charlson Comorbidity Index; CI, confidence interval; ET, essential thrombocythaemia; HCT, haematocrit; Hb, haemoglobin; PV, polycythaemia vera; RBC, red blood cell count; WBC, white blood cell count.

#### 3.1.1 Essential thrombocythemia

Among high‐risk ET patients, the most pronounced decline was observed for platelet counts. A typical 60‐year‐old female patient would start with an elevated platelet count of 604.6 × 10^9^/L (95% CI 497.0–735.5) at the index diagnosis and a statistically significant (*p* < 0.001) reduction to 337.7 × 10^9^/L (95% CI 278.0–410.1) would be observed by year 1. From there, average platelet counts slowly increased again to 436.6 × 10^9^/L (95% CI 330.6–576.7) by year 5 (Table 2 and Figure 1A). Consecutive platelet counts measured for the same patient could vary considerably between measurements, with 95% confidence intervals for a patient's plausible values ranging from 305.2 to 1,197.9 × 10^9^/L at index date (Figure 1A). Haematocrit and haemoglobin tended to decrease only marginally over the observation period, but we observed a drop in RBC count over the first year from 4.6 × 10^12^/L (95% CI 4.3–4.8) at index date to 3.9 × 10^12^/L (95% CI 3.7–4.2) at year 1 (*p* < 0.001), where it remained stable (Table 2 and Figure 1B–D). Average WBC count in the average patient similarly decreased 8.4 × 109/L (95% CI 7.2–9.7) at index to 6.8 × 109/L (95% CI 5.8–7.9) at year 1 (*p* = 0.005).

#### 3.1.2 Polycythaemia vera

Initially higher levels of FBC measurements around cohort entry were also observed in high‐risk PV patients (Table 2 and Figure 2). While the decline in platelet counts for a typical 60‐year‐old male patient with PV was less pronounced than in ET patients (Index: 323.8, 95% CI 290.7–360.6; Year 1: 268.8, 95% CI 242.1–298.5, *p* < 0.001), declines were particularly strong over the first month for both haematocrit and haemoglobin (Table 2, Figure 2A–C). Starting at the upper end of the normal range with 50.3% (95% CI 48.9–51.8) and 16.3 g/dl (95% CI 15.8–16.8) for haematocrit and haemoglobin respectively, typical values decreased to 45.2% (95% CI 43.7–46.7, *p* < 0.001) and 14.5 g/dl (95% CI 14.0–15.0, *p* < 0.001) by year 1. RBC counts similarly decreased from an average of 5.9 × 10^12^/L (95% CI 5.6–6.1) to 5.1 × 10^12^/L (95% CI 4.9–5.4, *p* < 0.001), whereas WBC counts decreased initially but remained well within the normal reference ranges.

### 3.2 Time spent in peripheral blood count remission

**FIGURE 3:**
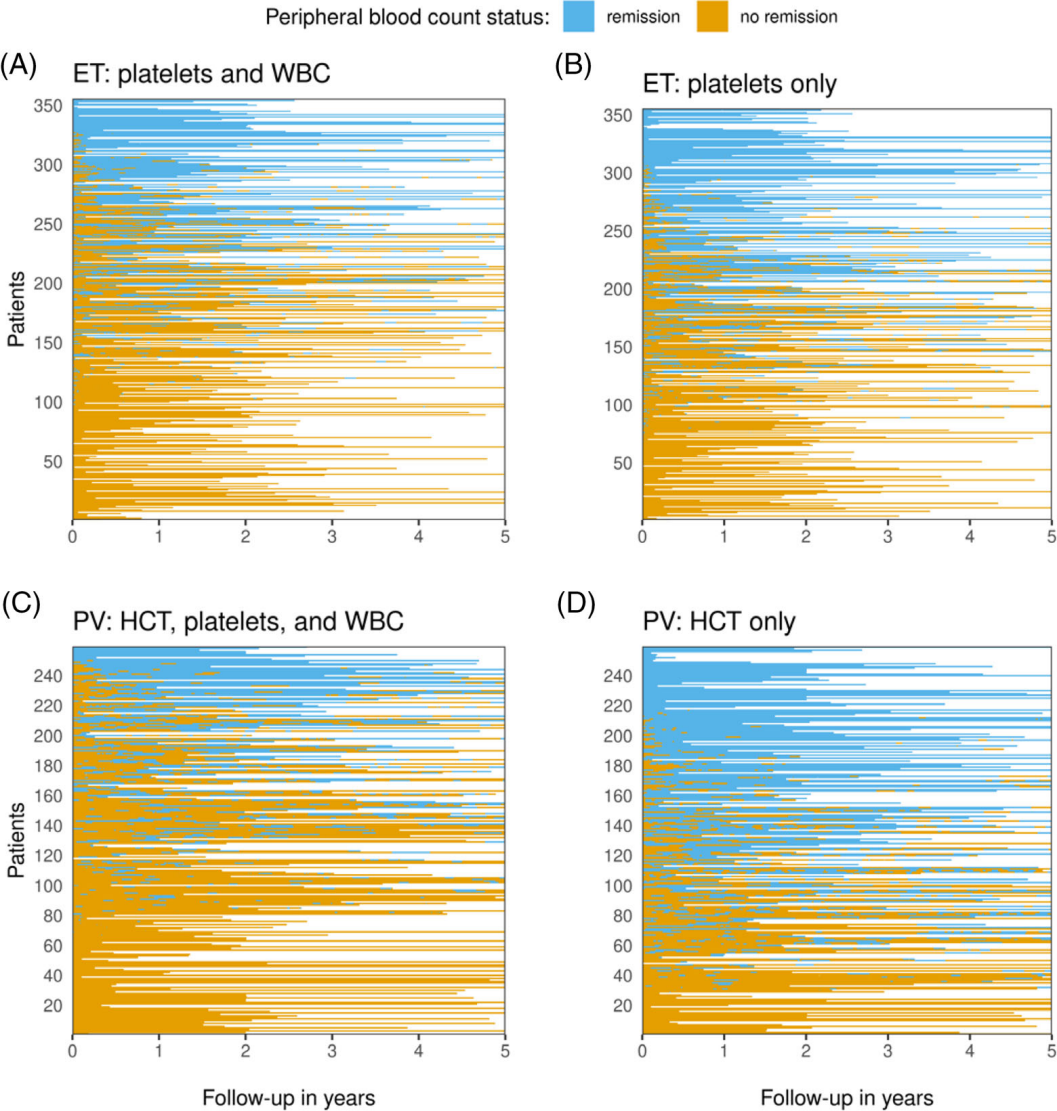
Follow‐up time spent in peripheral blood count remission according to European LeukemiaNet criteria for clinicohaematologic response for patients with ET and PV. Patients are ordered by the proportion of total observation time spent in remission. (ET – Essential thrombocythaemia, HCT– haematocrit, PV – Polycythaemia Vera, WBC – white blood cell count)

Overall, 301/374 (80.5%) high‐risk ET patients and 206/262 (78.6%) high‐risk PV patients with longitudinal blood count data achieved complete peripheral blood count remission at least once during their observation period. High‐risk patients frequently moved in and out of remission (Figure 3A–D). On average, high‐risk patients were in remission 39.2% (ET) and 29.1% (PV) of the follow‐up time respectively. This increased when considering haematological markers separately. ET patients achieved remission 46.4% of the observed time based on platelets alone (Figure 3B) and 76.3% based on WBC. Judging by haematocrit alone, PV patients were in remission 55.4% of the observed time (Figure 3D). This further increased to 71.5% for platelets and 69.0% for WBC.

### 3.3 Association between peripheral blood count remission and clinical outcomes

**FIGURE 4:**
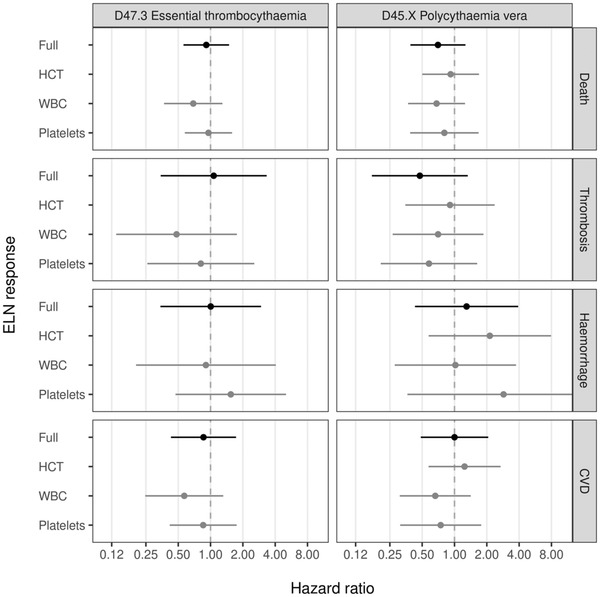
Association between achieving remission within 3 months of cohort entry in high‐risk ET or PV patients and risk of developing thrombosis, haemorrhage, other CVD, or dying of any cause. (CVD – cardiovascular disease, ET – Essential thrombocythaemia, HCT – haematocrit, PV – Polycythaemia Vera, WBC – white blood cell count)

During follow‐up, 68 deaths (95 per 1,000 patient‐years at risk [PY]), 13 thromboses (19 per 1,000 PY), 14 haemorrhages (21 per 1,000 PY), and 33 wider CVD events (53 per 1,000 PY) were observed among high‐risk ET patients. Among high‐risk PV patients, there were 49 deaths (68 per 1000 PY), 19 thromboses (29 per 1,000 PY), 13 haemorrhages (19 per 1,000 PY), and 31 wider CVD events (51 per 1,000 PY). Due to the relatively small number of events observed, no statistically significant associations were found at conventional levels where the 95% confidence limits are observed to cross the boundary of the null effect (Figure 4 and supplemental data in Tables 2 and 3). Reaching peripheral blood count remission was generally associated with reduced risks of developing the outcome. The only exception to this was haemorrhage, where patients that were in remission for haematocrit and platelets had a (not statistically significant) higher risk.

## 4 DISCUSSION

From our analysis, longitudinal changes in the haematological markers revealed reductions across all blood counts in the first year after baseline for both PV and ET patients, stabilising thereafter until year 5. This is most likely because of treatment, particularly for platelet counts in ET patients, which showed the largest statistically significant reduction, and in HCT, Hb and RBC in PV patients. Although comparative data in ET are lacking, the trends observed for PV in the first year are in agreement with a recent study by Ronner et al. [18]^,^ which found similar decreases in haematocrit levels, but relatively stable levels of platelets and leukocytes.

With regards to the proportion of PV patients achieving peripheral blood count remission, a similar trend was reported in previous studies [16]. Patients spent a larger proportion of time reaching thresholds for haematocrit (64%), leukocytes (86%) and platelet counts (71%), whereas patients spent just 45% of the observation period in complete remission. For ET patients, a relatively higher proportion (39.2%) of time was spent in peripheral blood count remission compared to PV (29.1%). However, there was a large difference between the proportion reaching the individual haematological thresholds, with just under 50% of the time spent under the platelet threshold, compared to around 80% of the time spent under the leukocyte threshold. Although the time spent in haematological remission was not detailed in previous studies, Carobbio et al. [13] indicated that 83% of patients were classified as in complete or partial remission at the 12‐month follow‐up. When looking at each haematological parameter individually, the number of patients achieving a platelet count of < 400 × 10^9^/L at 12‐months was just 26%, whereas those achieving a WBC count < 10 × 10^9^/L at 12‐months was 89%. This corroborates our finding that meeting the platelet target in routine care is more challenging than meeting the target leukocyte count in ET.

Although these findings highlight the challenge in achieving peripheral blood count remission in ET and PV in routine care, the extent to which meeting these thresholds translates to improved outcomes for patients is less clear. In agreement with previous studies of both ET and PV, our results highlight how those patients achieving complete remission within first 3‐months demonstrated no statistically significant reduction in the risk of thrombosis or bleeding [13, 14, 15, 16]. It should be noted in one study however, PV patients who were able to reach the platelet target count demonstrated a statistically significant reduction of between 30% and 90% in the risk of thrombosis or bleeding [16]. No statistically significant reduction in risk of either thrombosis or bleeding was observed for those achieving haematocrit or leukocyte targets. Likewise, whilst studies in ET did not show significant reductions in risk of thrombosis for those achieving platelet counts of < 400 × 10^9^/L, there was a significant reduction in thrombosis [13, 14] and mortality [14] for those who met the leukocyte threshold of < 10 × 10^9^/L.

A meta‐analysis conducted in 2019 by Carobbio et al. [19] also revealed that the presence of leucocytosis in ET and PV patients was associated with a statistically significant risk of bleeding and mortality. Leucocytosis may be a pertinent prognostic biomarker in chronic ET and PV despite its absence from the revised IPSET‐thrombosis criteria. Further longitudinal studies may help to better understand the relationship between leucocyte count and disease outcomes. A key aim of determining meaningful surrogate markers in PV and ET is in prognosticating thrombotic and disease transformations risk as well as overall survival. Other than a select few examples– and most of which are based on genomic modelling – there has been limited success in the creation and deployment of prognostic models in ET and PV [20, 21, 22].

## 5 STRENGTHS AND LIMITATIONS

The strengths of this study namely include the provision and retrospective analysis of longitudinal data in a large, real‐world UK patient cohort diagnosed with ET and PV, which is otherwise scarce in present literature. Our analysis utilises up to 20,000 observations from over 1,000 patients and explores the extent to which peripheral blood count remission targets are met over the course of the disease.

However, the study is not without its limitations. There are clinical and molecular similarities between PV and ET, which can lead to misrepresentation of disease pathophysiology and under‐ or overestimation of treatment effects [23]. The observational nature of the study did not make it possible to examine how specific treatments were associated with outcomes, however this study focuses only on those high‐risk patients reflecting the natural treated progression, where treatment is assumed to be based on best clinical practices as the time. The lack of recorded fluctuation in hematological markers after the first year of observation may also be attributed to the induction of apparent disease remission in these patients [2]. Coverage of JAK2 mutation status was limited and it's possible that those PV patients without a recorded JAK‐2 mutation status could be negative. Furthermore, the use of retrospective data from EHR means it is difficult to account for patients that may be lost to follow‐up, although the use of multilevel models would account for data attrition over time.

## 6 CONCLUSIONS

We have provided one of the first descriptions of real‐world data to show differences in laboratory haematological progression over a five‐year follow‐up period in high‐risk PV and ET patients. Additionally, we have provided a detailed overview of the extent to which peripheral blood count remission was achieved in those patients during a seemingly ‘stable’ period of the disease. This study highlights the challenges in achieving complete haematological remission. However, the analysis was unable to show any statistically significant increases in thrombotic or cardiovascular related risk for those patients not achieving remission within 3‐months.

## CONFLICT OF INTEREST

Mark Drummond received payment or honoraria by BMS for lectures, presentations, speakers bureaus, manuscript writing or educational events, expert testimony and support for attending meetings and/or travel in the last 36 months. Aurelie Duces and Gabrielle Emanuel are employees of BMS which funded the study. Adam Mead received grants, contracts or consulting fees from BMS. Adam Mead received payment for payment or honoraria by BMS for lectures, presentations, speakers bureaus, manuscript writing or educational events and support for attending meetings and/or travel in the last 36 months. Adam Mead participated on a BMS Data Safety Monitoring Board or Advisory Board. Sofia D'Abrantes was at Sensyne Health at the time of research but is now at AstraZeneca. No work has been carried out on this publication while working at AstraZeneca.

## ETHICS STATEMENT

The data were extracted, anonymised, and supplied by Chelsea & Westminster Hospital NHS Foundation Trust and Oxford University Hospitals NHS Foundation Trust in accordance with internal information governance review, NHS Trust information governance approval, and General Data Protection Regulation (GDPR) procedures outlined under the Strategic Research Agreement (SRA) and relative Data Sharing Agreements (DSAs) signed by both Trusts and Sensyne Health plc on 25th July 2018. Pursuant to the SRA, Sensyne commits to only process data in respect of projects that have identified patient benefit. As data were anonymised at source, ethical approval was not required.

## AUTHOR CONTRIBUTIONS

All authors designed the study. S.D and N.L carried out the data acquisition. All authors analysed and interpreted the data.

## Supporting information

Table S1. List of ICD‐10 codes used to define thrombosis, haemorrhage, and other cardiovascular disease.
Table S2. Incidence rate and age‐sex‐CCI adjusted hazard ratio of all‐cause mortality, thrombosis, haemorrhage, and CVD in 355 high‐risk ET patients, comparing patients that do and do not achieve ELN response criteria within 3 months.
Table S3. Incidence rate and age‐sex‐CCI adjusted hazard ratio of all‐cause mortality, thrombosis, haemorrhage, and CVD in 259 high‐risk PV patients, comparing patients that do and do not achieve ELN response criteria within 3 months.
