# Supplementary material for: Longitudinal trends of peripheral blood counts in polycythaemia vera and essential thrombocythemia patients in the UK
Source: EJHaem. 2022 Jun 29;3(3):785–93. doi: 10.1002/jha2.519 (PMC9421951; doi:10.1002/jha2.519)
Supplement: Supplementary file 1 — Table S1. List of ICD‐10 codes used to define thrombosis, haemorrhage, and other cardiovascular disease. Table S2. Incidence rate and age‐sex‐CCI adjusted hazard ratio of all‐cause mortality, thrombosis, haemorrhage, and CVD in 355 high‐risk ET patients, comparing patients that do and do not achieve ELN response criteria within 3 months. Table S3. Incidence rate and age‐sex‐CCI adjusted hazard ratio of all‐cause mortality, thrombosis, haemorrhage, and CVD in 259 high‐risk PV patients, comparing patients that do and do not achieve ELN response criteria within 3 months. [file JHA2-3-785-s001.docx]

**Manuscript title:** Longitudinal trends of peripheral blood counts in polycythaemia vera and essential thrombocythemia patients in the UK

**Short title:** Blood count trends in ET and PV patients

Lewis Carpenter^1^, Patrick Rockenschaub^1^, Grace B. Hatton^1^, Sofia D’Abrantes^1^, Edward Sims^1^, Nicholas Scott-Ram^1^, Aurélie Ducès^2^, Gabrielle Emanuel^2^, Adam J. Mead^3^, Mark W. Drummond^4^, Nadezda Lipunova^1^.

^1^ Sensyne Health, Oxford Science Park, Schrödinger Building, Heatley Rd, Oxford OX4 4GE

^2^ Bristol Myers Squibb Ltd, Uxbridge Business Park, Sanderson Road, Uxbridge, Middlesex, UB8 1DH

^3^ Medical Research Council Weatherall Institute of Molecular Medicine, John Radcliffe Hospital, Headington, Oxford, UK

^4^ Department of Haemato-Oncology, Beatson West of Scotland Cancer Centre, Glasgow, UK

**Supplemental data**

Supplementary Table 1. List of ICD-10 codes used to define thrombosis, haemorrhage, and other cardiovascular disease.

| **Condition** | **ICD-10 codes*** |
| --- | --- |
| Thrombosis | |
| Myocardial infarction | I21, I22, I23, I25.2 |
| Cerebral infarction | I63 |
| Stroke | G46.3, G46.4, G46.5, G46.6, G46,7, I64 |
| Transient Ischaemic Attack | G45.8, G45.9 |
| Deep vein thrombosis | I67.6, I80.1, I80.2, I80.3, I81, I82 |
| Pulmonary embolism | I26 |
| Other thrombosis | I51.3, I24, I74 |
| Haemorrhage | |
| Cerebral haemorrhage | I60, I61, I62.0, I62.1, I62.9, I63 |
| Anticoagulation defect | D66, D67, D68.0, D68.1, D68.2, D68.3, D68.4, D68.8, D68.9 |
| Purpura | D69 |
| GI haemorrhage | K25.0, K25.2, K25.4, K25.6, K26.0, K26.2, K26.4, K26.6, K27.0, K27.2, K27.4, K27.6, K28.0, K28.2, K28.4, K28.6, K29.0, K62.5, K92.0, K92.1, K92.2, R58 |
| Other haemorrhages | H11.3, H21.0, H31.3, H35.6, H43.1, H45.0, I78.0, I85.0, I98.3, S06.4, S06.5, S06.6 |
| Cardiovascular disease (including all above codes) | |
| Peripheral arterial disease | I73.1, I73.8, I73.9, I74.3, I74.4, I74.5 |
| Angina | I20.0, I20.1, I20.8, I20.9, I24.0, I24.8, I24.9 |
| Coronary heart disease | I25.0, I25.1, I25.3, I25.4, I25.5, I25.6, I25.8, I25.9 |
| Heart failure | I50, I11.0, I13.0, I32.2 |
| Cardiac arrest | I46.0, I46.1, I46.9 |

* 3-character codes include all 4-character subcodes, e.g. I21 includes I21.0-I21.9

Supplementary Table 2. Incidence rate and age-sex-CCI adjusted hazard ratio of all-cause mortality, thrombosis, haemorrhage, and CVD in 355 high-risk ET patients, comparing patients that do and do not achieve ELN response criteria within 3 months.

|  | Follow-up person-years | No. of events | Incidence rate (x 1000 person-years) | Hazard ratio * (age-sex-CCI adjusted) |
| --- | --- | --- | --- | --- |
| All-cause mortality | | | | |
| ELN response |  |  |  |  |
| Yes | 421.2 | 40 | 95.0 | 0.91 (0.56-1.49) |
| No | 295.0 | 28 | 94.9 | 1 |
|  |  |  |  |  |
| WBC count  < 10 x 10^9^/L |  |  |  |  |
| Yes | 624.7 | 56 | 89.6 | 0.69 (0.37-1.29) |
| No | 91.4 | 12 | 131.2 | 1 |
|  |  |  |  |  |
| Platelet count  < 400 x 10^9^/L |  |  |  |  |
| Yes | 462.6 | 44 | 95.1 | 0.96 (0.58-1.59) |
| No | 253.5 | 24 | 94.7 | 1 |
|  |  |  |  |  |
| Thrombosis | | | | |
| ELN response |  |  |  |  |
| Yes | 396.3 | 8 | 20.2 | 1.07 (0.34-3.33) |
| No | 287.0 | 5 | 17.4 | 1 |
|  |  |  |  |  |
| WBC count  < 10 x 10^9^/L |  |  |  |  |
| Yes | 594.8 | 10 | 16.8 | 0.48 (0.13-1.76) |
| No | 88.5 | 3 | 33.9 | 1 |
|  |  |  |  |  |
| Platelet count  < 400 x 10^9^/L |  |  |  |  |
| Yes | 437.4 | 8 | 18.3 | 0.81 (0.26-2.55) |
| No | 245.9 | 5 | 20.3 | 1 |
|  |  |  |  |  |
| Haemorrhage | | | | |
| ELN response |  |  |  |  |
| Yes | 398.4 | 8 | 20.1 | 1.00 (0.34-2.95) |
| No | 279.8 | 6 | 21.4 | 1 |
|  |  |  |  |  |
| WBC count  < 10 x 10^9^/L |  |  |  |  |
| Yes | 591.0 | 12 | 20.3 | 0.91 (0.20-4.05) |
| No | 87.2 | 2 | 22.9 | 1 |
|  |  |  |  |  |
| Platelet count  < 400 x 10^9^/L |  |  |  |  |
| Yes | 432.9 | 10 | 23.1 | 1.54 (0.47-5.05) |
| No | 245.3 | 4 | 16.3 | 1 |
|  |  |  |  |  |
| CVD | | | | |
| ELN response |  |  |  |  |
| Yes | 355.4 | 18 | 50.7 | 0.86 (0.43-1.73) |
| No | 267.7 | 15 | 56.0 | 1 |
|  |  |  |  |  |
| WBC count  < 10 x 10^9^/L |  |  |  |  |
| Yes | 541.5 | 26 | 48.0 | 0.57 (0.25-1.31) |
| No | 81.6 | 7 | 85.8 | 1 |
|  |  |  |  |  |
| Platelet count  < 400 x 10^9^/L |  |  |  |  |
| Yes | 389.6 | 20 | 51.3 | 0.85 (0.42-1.75) |
| No | 233.5 | 13 | 55.7 | 1 |

Supplementary Table 3. Incidence rate and age-sex-CCI adjusted hazard ratio of all-cause mortality, thrombosis, haemorrhage, and CVD in 259 high-risk PV patients, comparing patients that do and do not achieve ELN response criteria within 3 months.

|  | Follow-up person-years | No. of events | Incidence rate (x 1000 person-years) | Hazard ratio * (age-sex-CCI adjusted) |
| --- | --- | --- | --- | --- |
| All-cause mortality | | | | |
| ELN response |  |  |  |  |
| Yes | 278.5 | 18 | 64.6 | 0.70 (0.39-1.27) |
| No | 437.9 | 31 | 70.8 | 1 |
|  |  |  |  |  |
| Haematocrit 0.40 < x < 0.45L/L |  |  |  |  |
| Yes | 435.3 | 32 | 73.5 | 0.92 (0.50-1.69) |
| No | 281.1 | 17 | 60.5 | 1 |
|  |  |  |  |  |
| WBC count < 10 x 10^9^/L |  |  |  |  |
| Yes | 552.7 | 34 | 61.5 | 0.68 (0.37-1.25) |
| No | 163.7 | 15 | 91.6 | 1 |
|  |  |  |  |  |
| Platelet count < 400 x 10^9^/L |  |  |  |  |
| Yes | 575.8 | 40 | 69.5 | 0.81 (0.39-1.68) |
| No | 140.6 | 9 | 64.0 | 1 |
|  |  |  |  |  |
| Thrombosis | | | | |
| ELN response |  |  |  |  |
| Yes | 255.2 | 5 | 19.6 | 0.48 (0.17-1.33) |
| No | 401.3 | 14 | 34.9 | 1 |
|  |  |  |  |  |
| Haematocrit  x < 0.45L/L |  |  |  |  |
| Yes | 397.9 | 12 | 30.2 | 0.91 (0.35-2.37) |
| No | 258.7 | 7 | 27.1 | 1 |
|  |  |  |  |  |
| WBC count < 10 x 10^9^/L |  |  |  |  |
| Yes | 506.4 | 13 | 25.7 | 0.70 (0.27-1.86) |
| No | 150.1 | 6 | 40.0 | 1 |
|  |  |  |  |  |
| Platelet count < 400 x 10^9^/L |  |  |  |  |
| Yes | 526.6 | 14 | 26.6 | 0.58 (0.21-1.63) |
| No | 130.0 | 5 | 38.5 | 1 |
|  |  |  |  |  |
| Haemorrhage | | | | |
| ELN response |  |  |  |  |
| Yes | 261.3 | 6 | 23.0 | 1.30 (0.43-3.92) |
| No | 422.4 | 7 | 16.6 | 1 |
|  |  |  |  |  |
| Haematocrit x < 0.45L/L |  |  |  |  |
| Yes | 410.2 | 10 | 24.4 | 2.13 (0.57**-**7.93) |
| No | 273.4 | 3 | 11.0 | 1 |
|  |  |  |  |  |
| WBC count < 10 x 10^9^/L |  |  |  |  |
| Yes | 525.1 | 10 | 19.0 | 1.02 (0.28-3.74) |
| No | 158.5 | 3 | 18.9 | 1 |
|  |  |  |  |  |
| Platelet count < 400 x 10^9^/L |  |  |  |  |
| Yes | 546.7 | 12 | 21.9 | 2.86 (0.36-22.5) |
| No | 136.9 | 1 | 7.3 | 1 |
|  |  |  |  |  |
| CVD | | | | |
| ELN response |  |  |  |  |
| Yes | 235.5 | 13 | 55.2 | 1.00 (0.49-2.06) |
| No | 378.0 | 18 | 47.6 | 1 |
|  |  |  |  |  |
| Haematocrit x < 0.45L/L |  |  |  |  |
| Yes | 361.1 | 21 | 58.2 | 1.24 (0.57-2.69) |
| No | 252.4 | 10 | 39.6 | 1 |
|  |  |  |  |  |
| WBC count < 10 x 10^9^/L |  |  |  |  |
| Yes | 469.7 | 21 | 44.7 | 0.66 (0.31-1.42) |
| No | 143.9 | 10 | 69.5 | 1 |
|  |  |  |  |  |
| Platelet count < 400 x 10^9^/L |  |  |  |  |
| Yes | 488.3 | 24 | 49.2 | 0.74 (0.31-1.77) |
| No | 125.3 | 7 | 55.9 | 1 |
